# P10-07 m-Health apps and health-promoting behavior: medical students' opinion

**DOI:** 10.1093/eurpub/ckac095.146

**Published:** 2022-08-29

**Authors:** Nikoleta Leventi, Aleksandrina Vodenitcharova, Kristina Popova, Antoniya Yanakieva, Kremena Ivanova

**Affiliations:** 1 FPH, Dept HTA, MU-Sofia, Sofia, Bulgaria; 2 FPH, Dept Bioethics, MU-Sofia, Sofia, Bulgaria; 3 FPH, Dept. Social Medicine, MU-Sofia, Sofia, Bulgaria

**Keywords:** m-Health, digital health, health-promoting behavior

## Abstract

**Background:**

The recent COVID-19 pandemic showed us that the use of digital health technologies could contribute and make health systems and services more effective. People worldwide use digital health solutions to protect their health and promote well-being. WHO recognizes digital health as key to realizing better access to health for people and an important part of the future of health. The aim of the survey is to study medical students attitude towards m-Health applications installed on their devices, assess their usage, and identify their relation to students sport habits. The research as well focused on medical students intention to apply m-Health apps into their clinical practice in order to improve the quality of services provided to patients.

**Methods:**

In order to achieve the purpose of the study, an anonymous web based questionnaire was used among first year medical students in Medical University -Sofia in Bulgaria, and all the students participated voluntarily. Both the attitudes of the students towards m-Health application, and how sports habits are related with them were measured with closed questions. The study was conducted during January and February 2021. We used a descriptive statistical method to analyze the data.

**Results:**

Completed questionnaires were received from 133 students. Although the majority of the students (67%) have a positive attitude towards m-Health applications, only half of them (47%) believe that m-Health applications have affected (better and much better) their health-promoting behavior. Data about the use of m-Health applications into their future clinical practice shows that medical students will recommend these applications to their patients (60%), but less than half of them (39%) feel confident with the protection of their patients' personal data.

**Conclusions:**

The study was conducted to explore medical students' opinion about m-Health applications and the related health-promoting behavior. The results shows that although they are keen in using, and recommending m-Health applications, they still do not trust the treatment of the personal data. It is thus recommended that more efforts should be in the direction of m-Health applications trustworthiness development. Great acknowledge, and guidelines in this area are important to ensure the wider use of m-Health applications.

